# Literature Review of Pathogen Agnostic Molecular Testing of Clinical Specimens From Difficult-to-Diagnose Patients: Implications for Public Health

**DOI:** 10.1089/hs.2023.0100

**Published:** 2024-04-16

**Authors:** Diane L. Downie, Preetika Rao, Corinne David-Ferdon, Sean Courtney, Justin S. Lee, Shannon Kugley, Pia D. M. MacDonald, Keegan Barnes, Shelby Fisher, Joanne L. Andreadis, Jasmine Chaitram, Matthew R. Mauldin, Reynolds M. Salerno, Jarad Schiffer, Adi V. Gundlapalli

**Affiliations:** Diane L. Downie, PhD, MPH, is Deputy Associate Director for Science, Office of Readiness and Response; at the US Centers for Disease Control and Prevention, Atlanta, GA.; Preetika Rao, MPH, is a Health Scientist; at the US Centers for Disease Control and Prevention, Atlanta, GA.; Corinne David-Ferdon, PhD, is Associate Director of Science, Office of Public Health Data, Surveillance, and Technology; at the US Centers for Disease Control and Prevention, Atlanta, GA.; Sean Courtney, PhD, is a Health Scientist, at the US Centers for Disease Control and Prevention, Atlanta, GA.; Justin Lee, DVM, PhD, is a Health Scientist, Division of Global Health Protection; at the US Centers for Disease Control and Prevention, Atlanta, GA.; Shannon Kugley, MLIS, is a Research Public Health Analyst; in Social, Statistical, and Environmental Sciences, RTI International, Research Triangle Park, NC.; Pia D. M. MacDonald, PhD, MPH, is a Senior Infectious Disease Epidemiologist; in Social, Statistical, and Environmental Sciences, RTI International, Research Triangle Park, NC.; Keegan Barnes is a Public Health Analyst; in Social, Statistical, and Environmental Sciences, RTI International, Research Triangle Park, NC.; Shelby Fisher, MPH, is an Epidemiologist; in Social, Statistical, and Environmental Sciences, RTI International, Research Triangle Park, NC.; Joanne L. Andreadis, PhD, is Associate Director for Science, at the US Centers for Disease Control and Prevention, Atlanta, GA.; Jasmine Chaitram, MPH, is Branch Chief, at the US Centers for Disease Control and Prevention, Atlanta, GA.; Matthew R. Mauldin, PhD, is Health Scientists, Office of Readiness and Response; at the US Centers for Disease Control and Prevention, Atlanta, GA.; Reynolds M. Salerno, PhD, is Director, Division of Laboratory Systems; at the US Centers for Disease Control and Prevention, Atlanta, GA.; Jarad Schiffer, MS, is Health Scientists, Office of Readiness and Response; at the US Centers for Disease Control and Prevention, Atlanta, GA.; Adi V. Gundlapalli, MD, PhD, is a Senior Advisor, Data Readiness and Response, Office of Public Health Data, Surveillance, and Technology; at the US Centers for Disease Control and Prevention, Atlanta, GA.

**Keywords:** Diagnostic, Infectious diseases, Public health preparedness/response, metagenomics, Emerging threats

## Abstract

To better identify emerging or reemerging pathogens in patients with difficult-to-diagnose infections, it is important to improve access to advanced molecular testing methods. This is particularly relevant for cases where conventional microbiologic testing has been unable to detect the pathogen and the patient's specimens test negative. To assess the availability and utility of such testing for human clinical specimens, a literature review of published biomedical literature was conducted. From a corpus of more than 4,000 articles, a set of 34 reports was reviewed in detail for data on where the testing was being performed, types of clinical specimens tested, pathogen agnostic techniques and methods used, and results in terms of potential pathogens identified. This review assessed the frequency of advanced molecular testing, such as metagenomic next generation sequencing that has been applied to clinical specimens for supporting clinicians in caring for difficult-to-diagnose patients. Specimen types tested were from cerebrospinal fluid, respiratory secretions, and other body tissues and fluids. Publications included case reports and series, and there were several that involved clinical trials, surveillance studies, research programs, or outbreak situations. Testing identified both known human pathogens (sometimes in new sites) and previously unknown human pathogens. During this review, there were no apparent coordinated efforts identified to develop regional or national reports on emerging or reemerging pathogens. Therefore, development of a coordinated sentinel surveillance system that applies advanced molecular methods to clinical specimens which are negative by conventional microbiological diagnostic testing would provide a foundation for systematic characterization of emerging and underdiagnosed pathogens and contribute to national biodefense strategy goals.

## Introduction

Advanced molecular techniques have demonstrated utility in the identification of pathogens in difficult-to-diagnose clinical situations where infection is suspected, yet routinely available microbiological testing yields negative results.^1^ Recent examples include patients with meningitis and encephalitis,^2^ undiagnosed respiratory illnesses,^3^ prosthetic joint infections,^4^ and an international outbreak of acute severe hepatitis in children.^5^ Techniques, such as metagenomic next generation sequencing (mNGS) (also referred to as shotgun metagenomics), have been used for pathogen agnostic testing in research settings; however, these are not yet widely used as a routine diagnostic tool in clinical laboratories due to inequitable availability, variations in sensitivity, resource constraints, regulatory burdens, and concerns for contamination or detection of nonclinically relevant pathogens.^6,7^ These techniques are potentially underutilized as a strategy for the early detection of emerging or reemerging human pathogens. However, they could be used to strengthen clinical care and public health preparedness and response if used appropriately and more widely.

Even after extensive testing in clinical laboratories, up to 60% of patients with serious infections may not have a causative pathogen identified.^1,8^ Current microbiological methodologies and diagnostic workflows are often limited in providing accurate diagnoses because they largely rely on pathogen-specific tests and are not always timely when searching for rare and dysgonic pathogens that are difficult to culture. Regardless of the type of microbe, pathogens can be detected directly from clinical specimens using mNGS.^9,10^ mNGS can characterize all nucleic acid in any specimen type and can detect rare organisms that may otherwise be missed by conventional diagnostic assays.^11,12^ Furthermore, mNGS techniques have been used to increase diagnostic yield for patients with acute^2^ or subacute infections^4^ or who are immunocompromised.^13^

Although mNGS holds much promise in terms of the depth and breadth of its applicability, there are numerous challenges to expanding its usage in clinical scenarios. Barriers such as the high initial and maintenance costs; need for highly trained specialists to conduct, analyze, interpret, and collate testing; long turnaround time and elaborate work flows; variations in analytic sensitivity; and difficulties with result interpretation all represent significant challenges to widespread adoption.^14,15^ Additionally, there are multiple limitations in clinical pathogen detection, including the shortage of complete genomic sequences for certain organisms; the need for clear criteria, guidelines, and interpretive standards; and the decreased test performance for some specimens (eg, intraocular fluid, joint fluid).^14^ Furthermore, the difficulty of validating mNGS assays according to Clinical Laboratory Improvement Amendments (CLIA) regulations may limit the number of laboratories that can perform mNGS.

However, as usage of mNGS increases in the postpandemic era, the costs may decrease,^16^ and more comprehensive database libraries should enhance interpretation, sensitivity, and clinical translation. Additionally, the development of guidelines may provide a framework to define clinical utility and criteria.^15^ Despite these recognized challenges, mNGS has the potential to be a timely, unbiased pathogen agnostic approach to detection that does not depend on predetermined targets or culture techniques and can be deployed in pathogen agnostic clinical scenarios to identify novel emerging and reemerging pathogens.^1,6,7,14,15^

Biomedical literature offers a robust and deep source of critical information, which, when paired with other sources and selectively curated, can be used to identify academic collaborators, track the performance of emerging technologies, and develop best practices to support a national pathogen agnostic sentinel surveillance system to advance biosecurity. We conducted a targeted review of the published biomedical literature to improve the understanding of how mNGS is used in clinical, research, and pathogen surveillance activities. Findings from this literature review will increase understanding of the potential for coordinating data sharing and reporting from entities that perform these tests to support regional and national public health surveillance and provide insight regarding threats from emerging pathogens.

## Methods

For the purposes of this review, the term “metagenomics” was defined as a pathogen agnostic, unbiased, sequencing technique that can produce sequence data of all microorganisms found in each specimen. Additionally, 16S/18S/ITS amplicon-based sequencing was included due to its importance in surveillance for emerging pathogens.

A literature search and selection process were used to address the aims of this study. We searched for peer-reviewed literature published before February 2023 and selected illustrative examples (“exemplars”) of publications that reported notable discoveries using a pathogen agnostic approach on human clinical specimens.

For the National Library of Medicine National Center for Biotechnology Information PubMed literature database, we developed a search strategy that included articles published from 1966 to February 2023, using multiple keywords and phrases. Terms were organized using “OR” and “AND” operators to represent entities (“who”), activities (“how”), and findings (“what”) (see Table 1). The search focused almost exclusively on pathogen agnostic approaches from 2006 to February 2023, specifically mNGS, and was limited to infectious disease among humans. The initial search of PubMed was conducted in October 2022, and a final scan for relevant publications was performed in February 2023.

**Table 1.** PubMed Search Strategy

Dates	Search Strategy	Retrieval	Retained
October 2022	((“Shotgun sequenc^*^” OR “Deep learning” OR “Pathogen detection” OR “Pathogen agnostic” OR “Metagenomic sequenc^*^” OR “Sentinel surveillance” OR “Syndromic surveillance” OR “Ongoing detection” OR “testing ecosystem^*^” OR “novel biotechnolog^*^” OR “early warning” OR “wastewater epidemiology” OR “metagenomic next-generation”) AND (“partner network^*^” OR “response network^*^” OR “pathogen discovery” OR “BSL-3” OR “biosafety level 3” OR “biological safety level 3” OR “high containment laborator^*^” OR “Special Pathogens Laboratory” OR “centers for disease control”)) OR ((“Shotgun sequenc^*^” OR “Deep learning” OR “Pathogen detection” OR “Pathogen agnostic” OR “Metagenomic sequenc^*^” OR “Sentinel surveillance” OR “Syndromic surveillance” OR “Ongoing detection” OR “testing ecosystem^*^” OR “novel biotechnolog^*^” OR “early warning” OR “wastewater epidemiology” OR “metagenomic next-generation”) AND (“Unique microorganism^*^” OR “Novel pathogen^*^” OR “Potential outbreak^*^” OR “Emerging pathogen^*^” OR “Reemerging pathogen^*^” OR “outbreak preparedness” OR “Rare pathogen^*^” OR “Underdiagnosed pathogen^*^” OR “unknown pathogen^*^” OR “pathogen detection” OR “fastidious organism^*^” OR “novel polyomavirus^*^”))	4,308	93
February 2023	(“novel”[All Fields] OR “re emerg^*^”[All Fields] OR “undetect^*^”[All Fields]) AND “polyomavirus”[All Fields] AND “human”[All Fields] AND (“laboratory”[All Fields] OR “molecular”[All Fields] OR “sequenc^*^”[All Fields] OR “surveillance”[All Fields] OR “epidemiology”[All Fields])	320	11

The search resulted in an initial retrieval of more than 4,000 records. A formal, systematic screening of the initial literature retrieval was not feasible due to the volume of records retrieved. Given the overall exploratory objective of the review, an informal selection process was used to narrow the initial retrieval to a subset of publications that focused on pathogen agnostic approaches and were limited to infectious disease in humans. This resulted in a subset (n=104) that was representative of the main search terms. Additional publications were identified (n=56) through referrals by partners or through informal and exploratory searching including review of references, Google searches, and author searches. This subset was then further examined to identify a representative sample set—the “exemplars.” For the selection of exemplars, the search was limited to case reports, case series, and studies involving clinical trials, research, and surveillance activities. Exemplars had to include 1 or more human subjects or specimens obtained from humans experiencing illness and describe the use of mNGS to detect or identify 1 or more pathogens. Opinion and theoretical pieces and publications on foodborne illness surveillance and antimicrobial resistance were excluded. The review was limited to empirical studies to provide insight into practical and actual applications and use of the diagnostic approach (inclusion of nonempirical publications generally provides insight into themes or perspectives and requires a wholly different, qualitative, or thematic approach to synthesis). US and non-US-based publications were considered. A total of 34 exemplars were selected for data abstraction and summary.

We developed a study characteristics and content data extraction sheet in Microsoft Excel to capture data elements such as author names, clinical presentation, molecular techniques, study type, and laboratory protocols, as well as other data elements of interest (eg, patient immune status and travel history) as available. To supplement the entity analysis, information about funding sources was extracted from the funding and acknowledgments sections of the peer-reviewed articles.

## Results

Of the 34 exemplars, most publications were case reports or case series (n=21 publications, Table 2) while other studies involved prospective clinical trials, surveillance studies, research programs, or outbreak situations (n=13 publications, Table 3). Case reports were most often on single patients (n=17) or a series of patients (n=4) (Table 2). Metagenomic techniques were used to successfully identify pathogens directly from patient specimens, including cerebrospinal fluid,^13,17-25^ respiratory secretions, ^26-31^ skin and soft tissue or bone and joint tissues/synovial fluid,^32,34^ heart valve tissue,^35^ and urine.^36^ A review of the methods sections revealed significant heterogeneity in sensitivity and specificity of testing due to variability in study design, reference standards, clinical factors, and technical variation among the laboratories performing the testing. For exemplar US publications, laboratories of the University of California at San Francisco were featured prominently as testing laboratories.

**Table 2.** Peer-Reviewed Publications Using Pathogen Agnostic Testing Techniques (N=21)

Citation/Country	Clinical Presentation (No. Patients)	Molecular Technique/Laboratory Name	Pathogen(s) Identified (Known or Previously Unknown)	Laboratory Protocol or Program	Travel	Specimen Processing
*Central Nervous System Infection (n=10)*						
Fridholm H, et al (2016)^17^ Denmark	Encephalitis (1 patient)	Lawrence Livermore Microbial Detection Array (LLMDA) Statens Serum Institut, Copenhagen	Human pegivirus (previously unknown)	RNAseq library prepared and sequenced on Illumina platform; reads mapped to entire reference genome (Acc. nr. NC_001710); assembled sequence (Acc. nr. KP259281) clustered within genotype 2	Potentially (no travel outside of Scandinavia, bartender on cruise ship)	Serum and CSF tested negative on traditional assays, specimens sent to Statens Serum Institut for further analysis. After 8 days with severe neurological symptoms, the patient gradually recovered and was discharged from the hospital 4 weeks later for rehabilitation. Five weeks after discharge, she was still viremic for human pegivirus in serum, but viral load had decreased 21 times (Ct 27.8)
Mongkolrattanothai K, et al (2017)^18^ Mexico	Meningitis, encephalitis (1 patient)	mNGS UCSF Clinical Microbiology Laboratory (CLIA-licensed)	*Brucella melitensis* (known)	Library construction and analysis using a modified version of the SURPI (sequence-based ultra-literature pathogen identification) computational pipeline	Not reported	CSF specimen submitted for mNGS after patient was discharged from hospital; patient was contacted to return for treatment
Wilson MR, et al (2017)^13^ United States	Acute meningo-encephalitis (1 patient with an immune-compromising condition—renal transplant)	mNGS UCSF Center for Advanced Technology	West Nile virus (known)	Illumina Nextera; DASH, a novel molecular method for selectively depleting unwanted sequences from next-generation sequencing libraries	Not reported	Patient hospitalized for 41 days; CSF from initial lumbar puncture received for MDS on hospital day 19; sequencing library was prepared and put on an Illumina HiSeq 2500 27 days later (5 days post-hospital discharge); sequencing data available 2 days later (7 days post-hospital discharge); preliminary results reported to treating clinicians 31 days after receipt of the CSF specimen (9 days post-hospital discharge)
Piantadosi A, et al (2018)^19^ United States	Encephalitis (1 patient)	mNGS Not reported	Powassan virus (previously unknown)	Not reported	Not reported	mNGS performed on CSF collected on hospital day 5, plasma and whole blood from hospital day 4, and brain tissue from a biopsy performed on hospital day 11; patient enrolled in a research study, and mNGS of CSF performed on hospital day 8, identified Powassan virus within 96 hours
Wilson MR, et al (2018)^20^ United States	Subacute and chronic meningitis (7 patients)	mNGS UCSF Center for Advanced Technology	*Taenia solium*, *Cryptococcus neoformans*, human immunodeficiency virus-1, *Aspergillus oryzae*, *Histoplasma capsulatum*, *Candida dubliniensis* (all known, 1 with new site)	Statistical model leveraged mNGS data from water-only nontemplated control specimens and from patients with noninfectious neuroinflammatory syndromes to reduce false positives and false negatives	No travel	Not reported
Beck ES, et al (2019)^21^ United States	Relapsing meningitis (1 patient)	mNGS UCSF	*Taenia solium* (known)	Not reported	India (2009)	Not reported
Tschumi F, et al (2019)^22^ Switzerland	Lymphocytic meningitis, scrotal pain (1 patient)	Unbiased metagenomic sequencing Not reported	*Naples phlebovirus* (sandfly fever Naples virus; Toscana virus) (previously unknown)	NexteraXT protocol (Illumina)	Yes (4-day trip to Italy)	Tests and cultures inconclusive; specimen sent for unbiased metagenomic sequencing; reads were analyzed with VirMet, a dedicated bioinformatic pipeline
Cao J, Zhu XQ (2020)^23^ China	Consciousness disturbance, left hemiparesis, focal neurologic signs (1 patient)	mNGS Guangzhou Sagene Biotech	Human parvovirus B19 (known, new site)	Not reported	Not reported	Not reported
Zhou C, et al (2021)^24^ China	Fever, coma, pneumonia idiopathic thrombocytopenic purpura (1 patient)	mNGS Ingeni-Gen XunMinKang Biotechnology	*Nocardia farcinica* (known, new site)	High-throughput sequencing technology	Not reported	3-5 mL of CSF collected and sealed using a sterile technique, shipped on dry ice to laboratory to perform mNGS
Chen M, et al (2022)^25^ China	Acute meningitis-related symptoms; also had gastroenteritis (1 patient)	mNGS Not reported	*Porphyromonas endodontalis*, *Prevotella oris*, *Prevotella baroniae*, *Fusobacterium nucleatum*, *Streptococcus constellatus*, *Gemella morbillorum* (known, new site)	Not reported	Not reported	CSF from lumbar puncture and blood specimen collected and sent for mNGS and routine culture
*Respiratory Infection (n=6)*						
Li N, et al (2021)^26^ China	Pneumonia (3 patients)	mNGS Not reported	*Chlamydia psittaci* (known)	Sample processing and DNA extraction: aseptic processing, treated with enzymes within 24–48 hours of collection; selectively enriched using the Vision Medicals' Patho-NET; DNA extracted using the TIANamp Micro DNA Kit	Not reported	3 individuals from same family admitted to hospital; negative for all common pathogens of community-acquired pneumonia; BALF and sputum/blood specimens sent for mNGS
Yuan Y, et al (2021)^27^ China	Fever, paroxysmal cough, muscle soreness (1 patient)	Not reported	*Chlamydia psittaci* (known)	Not reported	Not reported	BALF, sputum, and blood cultures were negative during hospitalization; BALF and blood sent for mNGS
Huang J, et al (2022)^28^ China	Fever, cough, expectoration (1 patient)	mNGS Hugobiotech, Beijing, China; Vision Medicals, Guangzhou, China	*Eikenella halliae* (known, new site)	16S rRNA gene sequencing performed	Not reported	16S rRNA gene sequencing performed; pathogen was detected as *Eikenella* at the genus level; sputum specimen sent for mNGS to confirm species; previously negative mNGS data were reanalyzed and *Eikenella corrodens* was found in the background microorganisms. Pathogens detected by the 2 mNGS tests were different at the species level; high-throughput whole genome sequencing indicated *E. halliae*
Jiao M, et al (2022)^29^ China	Fever, cough, chest tightness, chest pain, dyspnea, local swelling (18 patients)	mNGS Not reported	13 different *Nocardia* species 8 other bacteria, 4 fungi, and 6 viruses	All specimens tested with microbial culture and mNGS	Not reported	Lung tissue, BALF, and CSF stored and transported in cryopreservation tubes on dry ice; blood specimens were collected in ethylene diamine tetraacetic acid blood collection tubes and transported with ice packs
Yan H, et al (2022)^30^ China	Pulmonary infection (1 patient)	mNGS Hugobiotech, Beijing, China	*Nocardia nova*, *Mycobacterium tuberculosis*, *Aspergillus fumigatus*, human cytomegalovirus (known)	Qualified libraries with different tags were pooled together and amplified and then sequenced with the NextSeq550 System (Illumina) for 150 cycles	Not reported	Sputum and BALF specimens sent several times for sputum smears and cultures; BALF specimens were submitted for PACEseq metagenomic next-generation sequencing; mNGS of the lung tissue revealed *N. nova* and human cytomegalovirus
Qin Z, et al (2022)^31^ China	Pulmonary infection (1 patient)	mNGS Clinical Genome Center, Guangxi Kingmed Diagnostics	*Cryptococcus neoformans* (known)	DNA extraction and library prep, single-end 75bp sequencing	Not reported	Samples taken for microbial culture and testing were contradictory; on hospital day 9, specimen was sent for mNGS testing
*Skin and Soft Tissue Infection, Bone and Joint Infection (n=3)*						
Lai SY, et al (2022)^32^ China	Initial presentation of cellulitis and skin abscess (1 patient with an immunocompromising condition—aplastic anemia)	mNGS Not reported	*Pseudomonas aeruginosa* from routine microbiology cultures, then by mNGS in BALF and CSF (known)	Not reported	Not reported	BALF and CSF specimens sent for traditional culture and mNGS at same time; mNGS findings returned within 24 hours
Gu H, et al (2022)^33^ China	Lumbar degeneration (1 patient)	mNGS Not reported	*Klebsiella aerogenes* (known)	Nextera XT DNA Library Preparation Kit (Illumina)	Not reported	Not reported
Huang C, et al (2023)^34^ China	Displaced prosthesis, periprosthetic osteolysis (1 patient)	mNGS, ptNGS V-Medical Laboratory (Guangzhou) Co., Ltd, Guangzhou, China (mNGS); Shanghai Pathogeno Medical Technology Co., Ltd., Shanghai, China (ptNGS)	*Coxiella burnetti* (known, new site)	Not reported	No travel	Samples collected after inflammatory tissue and abnormal fluid found in joint during surgery, conventional microbiological tests were negative
*Endocarditis (n=1)*						
Imai A, et al (2014)^35^ Japan	Infectious endocarditis (3 patients)	mNGS Not reported	*Streptococcus sanguinis*; *Enterococcus faecalis*; *Streptococcus mutans* (known)	Not reported	Not reported	Not reported
*Urinary Tract Infection (n=1)*						
Zhang M, et al (2021)^36^ China	Complicated urinary tract infection (1 patient)	mNGS, PMseq-DNA (BGI Genomics) Not reported	*Enterococcus faecium*, *Enterococcus hirae*, *Pseudomonas aeruginosa*, *Pseudomonas denitrificans*, *Candida albicans* (known)	Not reported	Not reported	Urine specimen collected for mNGS led to more targeted treatment for complicated urinary tract infection

Note: Articles are organized by primary clinical syndrome and year of publication.

Abbreviations: BALF, bronchoalveolar lavage fluid; CLIA, Clinical Laboratory Improvement Amendments of 1998; CSF, cerebrospinal fluid; Ct, cycle threshold; DASH, depletion of abundant sequences by hybridization; MDS, metagenomic deep sequencing; mNGS, metagenomic next-generation sequencing; Patho-NET, pathogen nucleic acid enrichment technology; RNAseq, RNA sequence; UCSF, University of California San Francisco.

**Table 3.** Exemplar Publications of Pathogen Agnostic Testing Using Advanced Molecular Techniques (n= 13)

Citation/Country	Clinical Presentation (No. Patients)	Molecular Technique/Laboratory Name	Pathogen(s) Identified (Known or Previously Unknown)	Program or Study	Laboratory Protocol	Context/ Comments
Gaynor AM, et al (2007)^3^ Multiple: Australia, United States	Acute respiratory tract infection symptoms Australia: 37/1,245; US cohort 1: 1/480; US cohort 2: 5/410	Shotgun sequencing Not reported	Human polyomavirus 4 (previously unknown)	Virology Laboratory at St. Louis Children's Hospital; Midwest Regional Center of Excellence for Biodefense and Emerging Infectious Diseases Research, Washington University St. Louis; Pilot Sequencing Program	Library construction, shotgun sequencing analysis, complete genome amplification, and sequencing described in the publication materials and methods section	Patient admitted to Royal Children's Hospital in Brisbane; NPA was collected in October 2003; PCR assays (n=17) for known respiratory viruses were negative
Yozwiak NL, et al (2012)^37^ Nicaragua	Dengue-like illness 123 undiagnosed specimens; 7 positive control specimens; virus sequence detected: 45/123	Virochip microarray and deep sequencing UCSF DeRisi Laboratory	Circovirus-like sequences, human herpesvirus 6, divergent sequences with similarity to sequences from viruses in the *Herpesviridae*, *Flaviviridae*, *Circoviridae*, *Anelloviridae*, *Asfarviridae*, and *Parvoviridae* families (known and divergent from known)	Ongoing pediatric dengue study in Managua, Nicaragua	Total nucleic acid from 140 mL of serum extracted using the QIAamp Viral RNA Isolation Kit (Qiagen), prepared a randomly primed dsDNA library that were fluorescently labeled and hybridized to Virochip arrays	Serum specimens collected from patients at the National Pediatric Reference Hospital in Managua, Nicaragua; tested for dengue virus at the Nicaraguan Ministry of Health; suspected dengue cases that were negative by diagnostic assays were included in the metagenomic analysis
Yu G, et al (2012)^38^ Multiple: Chile; Mexico; United States	Gastroenteritis 12/96	mNGS Not reported	MX polyomavirus (previously unknown)	Research protocols	Not reported	Specimens from existing repositories
Alquezar-Planas DE, et al (2013)^39^ Denmark	Upper and/or lower respiratory tract infection Among 500 cohort specimens, 92 otherwise negative specimens identified as HPIV4 using mNGS	Multiple: immunological, PCR-based assays, Roche Genome Sequencer (GS) FLX Titanium pyrosequencing (second and third generation sequencing) Not reported	Human parainfluenza virus type 4 (divergent from known)	Children ages 0 to 5 years hospitalized at Odense University Hospital for symptoms of acute respiratory infection	Viral particle purification and extraction, virome library construction, pyrosequencing and read analysis	No pathogen detected in 25% of specimens taken from hospitalized children presenting with respiratory infections; 92 nasopharyngeal specimens were processed using an in-house metagenomic high-throughput sequencing pipeline for viral detection
Rockett RJ, et al (2013)^40^ Australia	Healthy; symptomatic Respiratory: 38/1,385; blood: 0/161; CSF: 0/171; urine: 0/189; stool: 23/263	rtPCR; whole genome sequencing Not reported	*Deltapolyomavirus sextihominis*, human polyomavirus 9, *Trichodysplasia spinulosa*-associated polyomavirus, *Deltapolyomavirus decihominis* (previously unknown)	NA	Not reported	Specimens from existing repositories
Schlaberg R, et al (2017)^41^ United States	Community-acquired pneumonia Symptomatic: 53/70; Asymptomatic: 55/90	Multiple: NGS (RNAseq); panviral group PCR Not reported	Human parechovirus, human bocavirus, Epstein-Barr virus, human herpesvirus 6, human herpesvirus 7 (known)	CDC Etiology of Pneumonia in the Community (EPIC) study	RNA sequencing, library generation, and analysis of metagenomic data and panviral group PCR described in the publication methods	Specimens from children hospitalized with community-acquired pneumonia were collected within 72 hours of hospital admission; specimens were stored within 24 hours
Li T, et al (2019)^42^ Democratic Republic of the Congo	Fever, headache, diarrhea or vomiting, abdominal pain, fatigue, myalgia, bleeding 70/70	mNGS UCSF	*Zaire ebolavirus* (known)	Outbreak investigation	RNA was reverse transcribed with SuperScript III reverse transcriptase followed by second-strand DNA synthesis with Sequenase DNA polymerase; mNGS libraries constructed from amplified cDNA using the Nextera XT DNA Library Preparation Kit (Illumina). Multiplexed barcoded mNGS libraries sequenced as 150-bp paired-end runs	Blood specimens collected in Democratic Republic of the Congo; PCR tested locally; shipped to San Francisco, with some specimen degradation; tested at UCSF using PCR and mNGS
Saha S, et al (2019)^43^ Bangladesh	Neurologic infection, meningitis Idiopathic meningitis: 10/25; Bangladesh samples, CHIKV: 17/472	mNGS UCSF	*Salmonella enterica*, *Stenotrophomonas maltophilia*, *Bacillus cereus*, *Mycobacterium tuberculosis*, CHIKV, mumps virus, enterovirus B (known and previously unknown, new site)	Meningitis surveillance study supported by WHO conducted in Dhaka Shishu Hospital.	Raw sequencing reads using the IDseq Portal; initial alignment and removal of reads derived from the human genome performed using the Spliced Transcripts Alignment to a Reference (STAR) algorithm; BLASTn was used to extract all complete CHIKV genomes	Surplus CSF was stored at 4°C until it was transferred to >80°C, usually within 2 to 72 hours
Schubert RD, et al (2019)^44^ Multiple: Canada, United States (cases)	Acute flaccid myelitis Acute flaccid myelitis: 42; control: 58	mNGS Not reported	Non-polio enterovirus (known)	Enrollment in research studies or through public health surveillance (Boston Children's Hospital, UCSF, University of Colorado, California Department of Public Health) and the Division of Viral Diseases at the CDC	RNA sequencing libraries prepared using a previously described protocol, unbiased ultra-deep mNGS with an adaptation of the VirScan method for comprehensively detecting antiviral antibodies	Patient specimens were collected. CSF was shipped on dry ice to the laboratory and stored at −80°C until use
Lee RS, et al (2020)^45^ Canada	Tuberculosis 65 (50 from 2011-2012 outbreak, 15 older specimens from same village)	Deep sequencing, PacBio SMRT, Quant-iT PicoGreen dsDNA Assay McGill University/ Genome Québec Innovation Centre	*Mycobacterium tuberculosis* complex (known)	NA	DNA extraction using the van Soolingen method, genomic DNA quantified using the Quant-iT PicoGreen dsDNA Assay; to obtain depth of coverage needed (approximately 500–1,000 times for deep sequencing, compared to approximately 50–100 times as routinely done by public health) pooled libraries were run on 4 independent lanes	Tuberculosis outbreak in small village, sputum specimens taken for diagnosis, DNA from those specimens used to conduct deeper cluster analysis
Stelzer-Braid S, et al (2020)^46^ Australia	Uncomplicated hand, foot, and mouth disease, fever, myoclonic jerks or meningitis, encephalitis with or without cardiopulmonary failure 18/23	mNGS, full-length genome PCR+ sequencing Not reported	Enterovirus A71 (known and divergent)	NA	Viral nucleic acid was extracted using the QIAamp Viral RNA Extraction Kit Near full-length enterovirus genomes (approximately 7.4 kb; EV genome is between 7.2 kb and 8.5 kb in length) were amplified using Klentaq polymerase; annotated and trimmed sequences	Samples taken during epidemic of enterovirus at tertiary pediatric hospitals, tested by diagnostic laboratory at time of epidemic, then stored in Virology Reference Laboratories until the study
Zhou P, et al (2020)^47^ China	Pneumonia 7	mNGS Wuhan Institute of Virology	Severe acute respiratory syndrome coronavirus 2 (SARS-CoV-2) (previously unknown)	NA	High-throughput sequencing, pathogen screening and genome assembly reported in the publication methods section	Specimens from hospitalized patients collected and stored; specimens sent to Wuhan Institute of Virology for diagnosis of causative pathogen
Piantadosi A, et al (2021)^48^ United States	Meningitis, encephalitis 68 hospitalized patients with known (n=44) or suspected (n=24) CNS infections	mNGS and mNGS enhanced with hybrid captured and methylated DNA depletion Not reported	Human herpesvirus 6, varicella-zoster virus, West Nile virus, *Mycoplasma*, Powassan virus, *Borrelia burgdorferi*, *Anaplasma phagocytophilum*, enterovirus (echovirus 30 strains) (known and previously unknown)	The Prospective Encephalitis and Meningitis Study (PEMS) is a prospective cohort study enrolling adults who present to Massachusetts General Hospital with confirmed or suspected central nervous system infection.	Nextera XT DNA Library Preparation Kit (Illumina)	Enrolled patients with known/suspected central nervous system infections, collected specimens, sent for mNGS to identify pathogens

Note: Articles are organized by year of publication.

Abbreviations: CDC, Centers for Disease Control and Prevention; CHIKV, chikungunya virus; CSF, cerebrospinal fluid; HPIV4, human parainfluenza virus type 4; mNGS, metagenomic next-generation sequencing; NA, not applicable; NPA, nasopharyngeal aspirate; PCR, polymerase chain reaction; RNAseq, RNA sequence; UCSF, University of California San Francisco; WHO, World Health Organization.

A total of 13 publications were categorized as using metagenomic or traditional sequencing techniques (eg, targeted sequencing and fragment analysis) and performed for prospective clinical trials, surveillance studies, research programs, or outbreak situations (Table 2). These studies included patients with respiratory symptoms or infections (including tuberculosis), neurological syndromes, gastroenteritis, and fever syndromes. A study involving respiratory infections utilized knowledge from prior identification of a pathogen (eg, polyomavirus or human parainfluenza virus) that resulted in the screening of large numbers of specimens from patients with respiratory infections to look for those viruses.^39^ Another study involved identifying the etiology of pneumonia,^41^ a condition that is often managed without specific microbiological diagnoses. Several studies were noted to be conducting surveillance on patients with central nervous system infection syndromes where the focus was on attempting to identify a microbiological etiology for the infection.^43,48^ Several studies implemented advanced molecular testing in outbreak situations to identify a potential pathogen,^42,45^ including a study from China to identify the novel SARS-CoV-2 virus.^47^

Results from mNGS supported the identification of multiple new pathogens.^3,17,38,47^ Researchers used mNGS to detect single pathogens,^18,24,26,33,34^ multiple pathogens,^29-31,39,48,42^ and rare pathogens.^22,28^ Results from mNGS were also used to correlate with clinical symptoms,^42^ test specimens from patients with atypical or no symptoms,^21,40,49^ and facilitate more accurate^28^ and improved clinical care.^50^ Additionally, mNGS analyses yielded several significant detection advantages, including detection from a very ill or immunocompromised patient,^13,19,20^ when all other advanced tests and cultures performed were negative^27,32,33,35,50^ or inconclusive.^26,50^

Metagenomic sequencing was used to determine associations between genotype and clinical phenotype,^46^ elucidate etiologies,^23,41,43,44,46^ investigate a pathogen's epidemiology by subsequent whole-genome capture,^50^ and characterize clusters of divergent strains.^39^ Additionally, in some cases, mNGS offered a faster turnaround time for results than traditional culture methods^29^ and broader agnostic detection capabilities than nucleic acid amplification testing.^23,51^ Overall, mNGS exhibits good sensitivity and specificity for diagnosing central nervous system infection and diagnostic performance during clinical application by assisting in identifying the pathogen. However, the efficacy remains inconsistent, which warrants subsequent studies to further improve performance during clinical application.^49,52^ In some cases, diagnostic sensitivity was increased by more than 18% to 20%,^49,51^ without false positives,^51^ independent of specimen type,^53^ and without the need for viable or culturable pathogens.^35^ However, mNGS can be less sensitive than polymerase chain reaction for detecting very low-level infections,^48^ and as with other genomic methods, mNGS output still requires careful interpretation and substantial bioinformatics support.^45^

## Discussion

Advanced molecular methods, such as mNGS, can provide unbiased, pathogen agnostic testing for human clinical specimens. mNGS methods are available and can be leveraged to support clinicians and patients in identifying a potential pathogen in difficult-to-diagnose patients. However, it is not clear how widely and equitably these modalities of testing are available to clinicians and patients who would benefit from such testing. These techniques have the potential to drive significant improvements in patient care and health outcomes by facilitating microbiological diagnoses in patients whose underlying etiology may be difficult to identify using more conventional and readily available laboratory testing.^7,54^

The literature reviewed was generally robust regarding descriptions of techniques and information about testing of clinical specimens using mNGS. The literature review of exemplar publications identified 3 general types of pathogen detection: (1) clinical infections caused by previously known pathogens that were difficult to diagnose using conventional testing of patient specimens, (2) presence of previously known pathogens in a site that may represent a new manifestation of infection, and (3) potential new human pathogens that were previously unknown that may be causing the infection.

Broadly, there appear to be at least 4 clinical situations and scenarios where mNGS testing has been implemented: (1) individual patients with acute, subacute, and chronic clinical syndromes where infection is suspected, yet conventional and available microbiological testing yields no known pathogens—this may include identifying coinfections with pathogens in patients that may allow for targeted therapies; (2) diagnosing 1 or more patients who may present with a clinical syndrome that has recently been described to be caused by a new or previously unknown pathogen; (3) prospective clinical trials, retrospective case-control studies, surveillance studies, and research programs; and (4) outbreak situations where metagenomic sequencing is systematically implemented on patient specimens to establish microbiological diagnoses and understanding of the clinical syndrome.

From a review of the biomedical literature, it is not clear how most individual patients benefited from the advanced testing, as journal publications could take several months or years to prepare and publish in the public domain after peer review. Improving the timeliness of validated mNGS testing to clinicians could positively impact management of individual patients, in terms of ruling in known pathogens despite rare or new presentations or identifying coinfections with pathogens that could be treated with specific antimicrobial agents. This may have been especially important for patients with immunocompromising conditions. Similarly, for the publications describing clinical trials and surveillance studies, clinicians may have had access to near real-time results that could positively impact patient diagnosis and management. Either way, these publications play an important role in providing evidence for considering wider application of pathogen agnostic testing for certain clinical care situations and for sentinel surveillance to advance biosecurity.

With wider availability of metagenomic testing and improvements in the techniques themselves to overcome challenges, such as specimen contamination and interpretation of results, we envision several clinical and public health benefits of periodic reviews and incorporation of results from metagenomic testing on human clinical specimens. These include developing targeted diagnostic assays using mNGS identification of a potential pathogen in the early days of an outbreak or pandemic to increase the effectiveness of the clinical and public health response to emerging pathogens.^55^ Knowledge gained after an outbreak has subsided may also support understanding and preparation for subsequent outbreaks, including developing, incorporating, and deploying new and emerging pathogen-specific diagnostic targets into US Food and Drug Administration-approved multiplex testing panels, based on knowledge gleaned from metagenomic testing.^5^ Finally, systematic surveillance for emerging and reemerging pathogens in specific populations (eg, patients with immunocompromising conditions, nursing home patients, people who are incarcerated, persons experiencing homelessness, returning international travelers) may be sentinels for such pathogens.

Currently, there is no nationwide pathogen agnostic surveillance system for emerging or reemerging human pathogens in the United States. Developing a coordinated sentinel surveillance system that deploys advanced molecular methods to clinical specimens from patients whose specimens are negative by conventional diagnostic would provide a foundation for systematic detection of emerging, reemerging, and underdiagnosed pathogens. In addition, mNGS has the potential to identify bioengineered or novel naturally occurring strains of pathogens that may evade traditional diagnostics. If appropriately resourced and scaled after demonstrating utility and cost-effectiveness, such a system could significantly strengthen proactive preparedness and response capabilities and be responsive to national biodefense strategy goals.^56^

Coordination and integration between systems supporting frontline clinicians and local, regional, and federal government are important components of a nationwide surveillance system that successfully monitors for, identifies, and responds to emerging and reemerging pathogens. Such a surveillance system would need to be equitably distributed across the country with availability at local and regional levels, and it should be agile enough to provide support for specific and vulnerable populations. Therefore, ongoing detection and discovery for surveillance of emerging and reemerging human pathogens are central to public health preparedness and outbreak detection and response at regional, national, and international levels.

The literature review has inherent limitations, such as publication bias because negative results are rarely published, delays in publicly reporting potential new biothreats, and reliance on publicly available information. The literature review alone leaves gaps in understanding of processes, pipelines, and key details about the mechanics of how specimens are identified, transported to specialty laboratories, and tested. Published information was not always able to clarify whether the mNGS tests were validated by the laboratory, whether the tests were regulated and approved through CLIA, thus allowing for reporting to clinicians and patients, or whether results were used for reflex testing with CLIA-approved assays. The search was not a systematic review, which likely resulted in an incomplete list of recent publications that may potentially impact findings. Additionally, the time needed to prepare and submit research articles for publication in a peer-reviewed journal creates a temporal gap that limits the usefulness of the information for literature response in a real-world setting. This review did not specifically target preprint servers.

There may also be institutions and practitioners who use advanced molecular testing but do not publish their results or findings; these are most likely small-scale, personal professional networks that play a role in routing point-of-care patient specimens for metagenomic testing, or the growing field of private commercial entities offering advanced molecular testing. Furthermore, some agencies and institutions may be unable to publish in scholarly outlets (eg, national security programs, commercial entities, public health agencies). Finally, this review does not account for the vast literature and experience available in the One Health arena where pathogens are routinely discovered in animal reservoirs.^57,58^

Understanding the extent of the availability of such testing modalities to clinicians across the country with respect to academic, clinical, public health, and commercial laboratories is necessary. It is also important to understand the flow of patient specimens from the primary point of microbiological testing to laboratories that perform these specialized tests, the status of validation and CLIA-approval for these tests, the exchange of patient information and clinical details along with the specimen, and mode of payment (eg, insurance coverage) for these tests. Reflex testing with CLIA-approved clinical diagnostic assays is also required to inform patient care. It is essential to understand the equitable availability of these tests for patients who would most benefit across geographical, racial and ethnic, and social demographic barriers. As these technologies improve and are more widely used, consistent and standardized policies and guidelines are critical for clinicians, researchers, and public health professionals to recognize the limitations of these techniques, protect patient privacy, ensure accurate interpretation of results, and support the appropriate use of the results to benefit individual patients and public health.^54,55^

## Conclusion

Advanced pathogen agnostic testing using metagenomic sequencing is being performed in a limited number of settings to directly support patient care, and for research and surveillance purposes. Improved understanding of the current usage and availability of advanced molecular testing techniques in a variety of healthcare settings that directly provide care to patients with pathogen agnostic testing could help inform public health surveillance and intervention strategies. Overcoming limitations and barriers to wider adoption of advanced molecular methods for testing clinical specimens would provide a foundation for systematic characterization of emerging pathogens. Additionally, deploying advanced molecular methods to test clinical specimens from patients whose specimens are negative by conventional microbiological diagnostic testing can contribute to a more coordinated sentinel surveillance system and is responsive to national biodefense strategy goals.
